# Winter is coming: Food web structure and seasonality in a subtropical freshwater coastal lake

**DOI:** 10.1002/ece3.3031

**Published:** 2017-05-17

**Authors:** Ignacio Peralta‐Maraver, Anne L. Robertson, Enrico L. Rezende, Aurea Luiza Lemes da Silva, Denise Tonetta, Michelle Lopes, Rafael Schmitt, Nei K. Leite, Alex Nuñer, Mauricio M. Petrucio

**Affiliations:** ^1^ Department of Life SciencesRoehampton UniversityLondonUK; ^2^ Departamento de EcologíaFacultad de Ecología y Recursos NaturalesUniversidad Andres BelloSantiagoChile; ^3^ Department of Ecology and ZoologyFederal University of Santa CatarinaFlorianópolisBrazil; ^4^ Department AquacultureFederal University of Santa CatarinaFlorianópolisBrazil

**Keywords:** aquatic systems, community structure, top‐down regulation, trophic interactions

## Abstract

Food web studies provide a useful tool to assess the organization and complexity of natural communities. Nevertheless, the seasonal dynamics of food web properties, their environmental correlates, and potential association with community diversity and stability remain poorly studied. Here, we condensed an incomplete 6‐year community dataset of a subtropical coastal lake to examine how monthly variation in diversity impacts food web structure over an idealized time series for an averaged year. Phytoplankton, zooplankton, macroinvertebrates, and fish were mostly resolved to species level (*n* = 120 trophospecies). Our results showed that the seasonal organization of the food web could be aggregated into two clusters of months grouped here as ‘summer’ and ‘winter’. During ‘winter’, the food web decreases in size and complexity, with the number of trophospecies dropping from 106 to 82 (a 22.6% decrease in the number of nodes) and the trophic interactions from 1,049 to 637 between month extremes (a 39.3% drop in the number of links). The observed simplification in food web structure during ‘winter’ suggests that community stability is more vulnerable to the impact of any change during this period.

## 1 Introduction

Food web studies are an integrative way to explore ecosystems in which species interactions have a role that is as important as that of community composition (Bascompte, 2009). They are also fundamental for describing and quantifying ecosystem complexity (Dunne, 2009; May, 1972; Montoya, Pimm, & Solé, 2006), knowledge that is essential to predict and mitigate the consequences of global change (Ings et al., 2009). Food web properties, as complexity measures, incorporate the number of nodes (taxa and trophic resources) and links (trophic interactions between nodes) in the network (Thompson et al., 2012), whereas topology or architecture is mainly determined by the distribution of trophic links between species (link pattern and identity) (Montoya & Solé, 2003). Nevertheless, even though food web properties and their relationships have received considerable attention; studies dealing with the distribution of trophic links (link pattern) are rare (Montoya & Solé, 2003). Hence, reliability and predictability of food web models are dependent on the ability of researchers to identify the components and trophic interactions of the community under study. This is a challenging task because we have a very limited taxonomic knowledge of most biological communities (Mayr, 1998). Many researchers studying food webs are forced to work at higher taxonomic levels or to use trophospecies classifications, with greater effort directed at the higher trophic levels than at the base of the web (Ings et al., 2009) even though taxonomic resolution affects the value of complexity descriptors in food webs (Thompson & Townsend, 2000).

Furthermore, most studies do not include the whole, or even the majority, of the community to build the food web. Although these studies provide important information for certain groups of an assemblage, they are of limited use in the assessment of food web theory at a community level (Tavares‐Cromar & Williams, 1996). For example, Sánchez‐Hernández, Cobo, and Amundsen (2015) highlighted that relatively few studies on food web properties in lakes have included benthic organisms and that most of them have focused on the pelagic zone. Thus, existing comparison between communities based on food web models may be affected by artifacts caused by the limitations of the data on which they are based (Cohen et al., 1993; Polis, 1991; Winemiller, Pianka, Vitt, & Joern, 2001). Additionally, many analyses of food web properties focus on a single point in time and neglect the seasonal dynamics, even though it is broadly accepted that ecosystems are highly heterogeneous both in space and in time (Kolasa & Rollo, 1991; Levin, 1992; Stewart, John, & Hutchings, 2000). Therefore, temporal dynamics also remains a poorly understood feature of food web ecology (Thompson et al., 2012). A few studies have reported that food web structure may change as a result of intra‐annual variability in freshwater communities (Tavares‐Cromar & Williams, 1996; Thompson & Townsend, 2000), but the relationship between this seasonal dynamic of food web properties and link pattern with community biodiversity and environmental factors has not been addressed. These changes in food web properties and link pattern over time may occur as a result of oscillations in the composition and density of the organisms that compose the communities. This is especially important in freshwater ecosystems where community characteristics vary greatly over time due to the extraordinary diversity of life strategies that the organisms have (i.e., generation times and different hatching and emergence period in insects, seasonal blooms in algae) (Brönmark & Hansson, 2005; Giller & Malmqvist, 1998; Peralta–Maraver, López–Rodríguez, & Tierno de Figueroa, 2016), which are conditioned by a wide range of environmental factors (Lancaster & Downes, 2013; Olden, Poff, & Bestgen, 2006; Sand‐Jensen, 1989).

Although interest in the study and analysis of food web properties has increased considerably over recent decades (Sánchez‐Carmona, Encina, Rodríguez‐Ruiz, Rodríguez‐Sánchez, & Granado‐Lorencio, 2012), there are important questions that remain unanswered: What is the variation in food web properties and link pattern in natural communities over time? Does the variation in food web properties, based on the number of nodes and links, reflect the variation in the link pattern? What is the relationship between the seasonal dynamic of food web properties with community biodiversity and environmental factors? Therefore, the objectives of this work are (1) to analyze how the biodiversity and the food web properties and link pattern change monthly during the year in a freshwater coastal lake and (2) to determine how the seasonal gradient of biodiversity and environmental factors are related to the food web properties. In order to address these objectives, we used a two‐stage approach. Firstly, we built binary qualitative food webs to assess clustering of the community throughout the year based on complexity and topology measures. Secondly, we compared the resultant clusters in terms of community density, diversity, and composition. We reconstructed the whole community (both benthic and pelagic organisms from the littoral and pelagic zone) at a high taxonomic resolution from an incomplete long‐term dataset in a freshwater subtropical lake.

## 2 Methods

**Figure 1:**
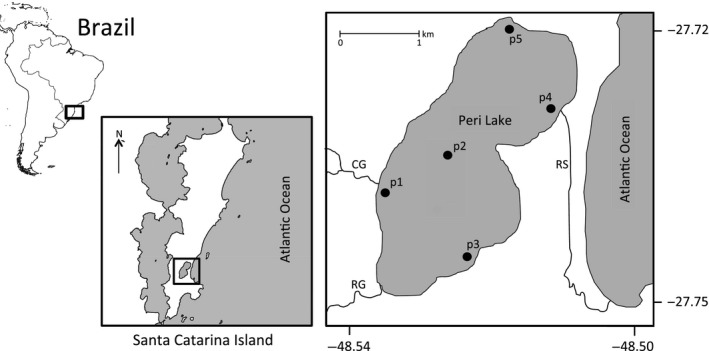
Study system Peri Lake and the different sampling sites (p1–p5). Inflowing streams Cachoeira Grande (CG) and Ribeirão Grande (RG), and outflowing Rio Sangradouro (RS) are also shown

The study was performed in the shallow coastal Peri Lake, located in Santa Catarina State, Southern Brazil (27°44′S and 48°31′W). Peri Lake has a surface area of 5.07 km^2^, maximum depth of approximately 11 m and an average depth of 4 m (Figure 1). It is a freshwater lake with conductivity generally below 70 μS/cm, separated from the sea by a 3 km long and 0.5 km wide sandbar to the East, while surrounded by 250–500 m mountains to the north, south, and west areas. The drainage basin is approximately 20 km^2^, and most of it is within a protected area with limited human influence and occupation. Two main streams (Cachoeira Grande and Ribeirão Grande) discharge into the lake, coming from the forested mountains. Peri lake is a nonstratifying water body and well mixed as a result of coastal winds (Hennemann and Petrucio, 2011).

Environmental factors (conductivity, pH, dissolved organic carbon, water temperature, total phosphorus, dissolved oxygen, total nitrogen, nitrogen–phosphorus ratio and water level) were measured in pelagic and littoral zones. Water samples were collected monthly at different locations and water depths (Figure 1) between 9:00 and 11:00 a.m. (local time, UTC/GMT—3 hr), from January 2008 to December 2014. The average value for each month was employed to construct one representative standardized year (Table S1). Water temperature, pH, conductivity, and dissolved oxygen were recorded with a calibrated probe (model YSI‐85), and dissolved organic carbon was determined in a TOC analyzer (Shimadzu TOC–5000A). Three liters of lake water were collected at each sampled site (Figure 1), and filtered through glass fiber filters (0.7 μm, Whatman GF/F) for extraction of chlorophyll a with 90% acetone, corrected for pheophytin (Lorenzen, 1967). Total nitrogen and phosphorus were determined from unfiltered waters according to Valderrama (1981), and alkalinity was determined through Gran titration (Mackereth, Heron, & Talling, 1978). Rainfall data were obtained from ICEA (Instituto de Controle do Espaço Aéreo, located 5.5 km from Peri Lake), while water level data were provided by CASAN (Water and Sanitation Company).

Water samples for phytoplankton and zooplankton determination were collected in the central part of the lake (Figure 1, p2) with a Van Dorn sampler at four depths according to the light penetration (for further details see Tonetta et al. 2013). Phytoplankton samples were collected monthly between July 2009 and March 2014 and preserved with formalin (final concentration 1.6%); aliquots of collected water were analyzed using an inverted microscope, where 400 individuals (cell, filament or colony) per sample were counted and identified. Zooplankton samples were collected between April 2011 and March 2012; water samples were filtered using a plankton net (50 μm mesh size), carbonated water was added to decrease the contraction of bodies, and samples were then fixed with 4% formaldehyde in the final concentration. We did not sample organisms smaller than 50 μm, and so excluded ciliates although we recognize that they may be important grazers. For quantification of rotifers and copepod nauplii, subsamples of 1 ml were counted using a Sedgwick–Rafter chamber under an optical microscope, whereas the quantification of cladocerans and copepods was done in petri dishes under a stereomicroscope. Macroinvertebrates were collected monthly between March 2008 and April 2009 at three sites (coastal zone, deeper waters, and center) of the lake. A total of 20 samples were collected at each site on each sampling occasion using an Eckman–Birge grab (15 × 15 cm, 0.025 m^2^ area); macroinvertebrates were preserved in 70% ethanol, counted, and identified. Finally, fish were sampled bimonthly between April 2008 and April 2012. Sampling was carried out in five stations (Figure 1), using gill nets with different mesh sizes (diagonal of the stretched square holes of 1.5; 2.0; 2.5; 3.0; 4.0; 5.0; and 6.0 cm) and a standard size of 20 × 1.5 m (150 m^2^ area). Nets were installed from 5:00 p.m. until 8:00 a.m., catches were identified, measured (length in mm and weight in g) in situ, and fixed for gut content analysis.

### 2.1 Community diversity and trophic interactions

Taxa were identified to the lowest possible taxonomic level (in most cases, nodes were identified at the species level, see List of identified organisms; Appendix S1). To study seasonal patterns in biodiversity in an idealized averaged year—that is, accounting for the different sampling periods—we calculated Shannon–Wiener′s diversity index (H′) monthly for each group (phytoplankton, zooplankton, macroinvertebrates, and fish). This index is a useful tool for following changes in relative density in a large number of species over time (Porter, 1977; Sager & Hasler, 1969). Specifically, H′ was calculated as:

$$H^{\prime }=-\sum_{i=1}^{S}P_{i}\ln\left(P_{i}\right)$$

where *S* is the number of taxa in the community, and P_*i*_ is the proportion of individuals in the community that belong to taxa *i* (Begon, Townsend, & Harper, 2005).

We then constructed a binary food web for different months using a qualitative interaction matrix between consumers and prey/resources (Supporting information; Appendix S2). The trophic interactions between organisms (links) were constructed on the basis of the gut content analysis for chironomidae larvae and fish, while it was extrapolated from the literature for macroinvertebrates and zooplankton (Diet list references; Appendix S1). The origin of the links (literature or gut content analysis) is available as part of the row data in Appendix S2. Chironomid larvae are usually the dominant and richest invertebrate group in freshwater benthic habitats (Ferrington, Berg, & Coffman, 2008). Hence, it could be assumed as a good model group to infer general ecological patterns of invertebrates. Chironomidae trophic interactions were studied by mounting specimens on semi‐permanent slides. At least 10 identified individuals (up to 30) were analyzed per period. Contrary to the rest of macroinvertebrates, this technique was really successful with chironomidae due to the small size (5–15 mm) and semi‐transparent soft body of larvae. For each sampling period, the gut contents of up to five individuals of each species of fish were dissected to determine their trophic interactions. Incorporating species interactions from published literature, despite its limitations, has been widely used in ecological studies of food webs as the best strategy to avoid excessive sampling effort (i.e., Layer, Hildrew, Monteith, & Woodward, 2010; Piechnik, Lawler, & Martinez, 2008; Pocock, Evans, & Memmott, 2012; Sánchez‐Hernández et al., 2015; Strong & Leroux, 2014). For those species whose feeding habits were not available, published information of similar taxonomic level (genus or family level for those organisms identified to species level) was employed because related species are likely to share similar traits, such as body size, feeding mode, and habitat preference (Eklöf, Helmus, Moore, & Allesina, 2012). Unfortunately, studies on ontogenetic changes in the diet of subtropical invertebrates are very rare. Therefore, intraspecific seasonal dietary shifts could not be included for links extracted from the literature and these could be underestimated in some cases. However, in order to assess the accuracy of our methodology, the pattern in total number of links was compared throughout the year with the subset of links identified from the gut content analysis.

### 2.2 Statistical analyses

Because there was not a single sampling scenario in all cases (Fig. S1), an idealized time series of species composition, abundance, and food web structure was constructed from 2008 to 2014. Following Boit, Martinez, Williams, and Gaedke (2012), we pooled monthly species composition during the whole study period, we employed monthly averages of organism's abundances in our analyses, and we constructed the food webs based on the cumulative knowledge of the links. While it is true, this methodology underestimates potential interannual variation in abundance of organisms (especially macroinvertebrates and zooplankton in this study), it may be expected that community composition and qualitative structure of the food web show little year‐on‐year variation (i.e., assuming the nonextinction of species in this protected area during the studied period). Anyhow, the nonexistence of dramatic changes in composition was assessed for fish and phytoplankton. To do so, similarity in fish and phytoplankton composition was compared between years using community data matrix (as 1—Sørensen dissimilarity index). The Shannon–Wiener′s diversity index (introduced above) was calculated using a Bayesian multinomial model. This approach allowed us to include all the incomplete information we had of the community and then obtain the pooled H^′^ and its uncertainty from the posterior distribution (McCarthy, 2007). A multinomial distribution was used for modeling the proportion of individuals belonging to each taxa based on the sampled data, and uninformative Dirichlet distribution was used as prior in the model. Model fitting was performed using a Markov chain Monte Carlo (MCMC) sampling procedure, constructing the posterior estimates of plausible biodiversity values and credibility intervals. 5,000 iteration with a burn‐in of 1,000 was used ensuring that the chain reached its stationary distribution. The Bayesian multinomial model and the MCMC sampling process were carried out with the free software openBUGS 3.0.7 for Microsoft (Spiegelhalter, Thomas, Best, & Lunn, 2003).

We employed two separate approaches to determine how food web structure varied throughout the year. First, we employed a NMDS ordination model to compare the dissimilarities in 16 qualitative network descriptors such as, for example, number of nodes, link density and connectance, across months (the list and explanation of these properties are available in Supporting information; Table S2). This is an effective method for the ordination of ecological data that works with rank orders (rather than absolute values) and can handle nonlinear responses of the biological attributes of any shape and effectively and robustly find the underlying gradients (Oksanen, 2015; Quinn & Keough, 2002). In order to reduce excessively large differences between smallest nonzero abundance and largest abundance, the variables were transformed using Wisconsin double standardization (Bray & Curtis, 1957). This transformation improves the gradient detection ability of similarity index (Oksanen, 2015). Then, a Bray–Curtis similarity index was used to build the distances matrix between months, and the ordination was reduced to two dimensions with the NDMS. Because the ordination provided by the NMDS model depends on the starting configurations of the communities in the multidimensional space, we ran the model iteratively to find the ordination with the best goodness of fit (Oksanen, 2015).

Subsequently, environmental factors, density, and diversity values by group (phytoplankton, zooplankton, macroinvertebrates, and fish) were fitted to the NMDS model following López‐Carretero, Díaz‐Castelazo, Boege, and Rico‐Gray (2014), and the association between these variables and the ordination was assessed by comparing the model of pairwise interactions with 1,000 permutations of a given null model. Because pelagic and littoral measurements provided qualitatively identical results, here we report fitted results for environmental correlates in the littoral zone.

Second, we assessed the topological consistency of the food web throughout the year, estimating the similarity in link patterns between months (as 1‐ Sørensen dissimilarity index). Whereas the NMDS analysis clusters months based on food webs with similar numerical descriptors and informs on the environmental factors that are correlated with observed clusters, this analysis ensures that structural similarities reflect the consistency in trophic interactions across months. Consistency in the resulting cluster of month from both methods was compared to assess whether the variation in food web properties, based on the number of nodes and links, reflected the variation in the link pattern.

Finally, we compared the 16 food web properties, density, and diversity of the communities between the obtained clusters of months (from NMDS and topological similarity analysis) using regular Kruskal–Wallis rank‐sum tests. All analyses were performed with the Vegan package (Oksanen et al., 2013) within the R software platform (R Core Team 2014).

## 3 Results

### 3.1 Food web structure

**Figure 2:**
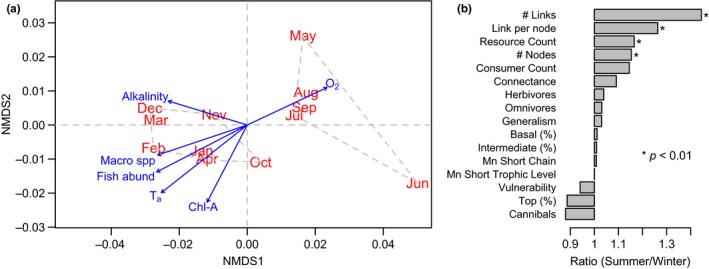
(a) NMDS ordination model of the community food web by month based on 16 network properties, overlapped with those environmental correlates and community descriptors that were significantly correlated (*p* < .05) with the ordination. The arrows depict the direction and magnitude of the seasonal gradient. (b) Seasonal effects on network properties, expressed as the ratio between mean estimates for summer and winter. Asterisks denote significant differences based on Kruskal–Wallis rank‐sum test

The NMDS ordination model based on 16 food web properties (Table S2) was run 100 times for the two‐dimensional ordination with a very high goodness of fit between the distances in the ordination against the original data (linear fit R^2^ = .995, nonmetric fit R^2^ = .990). Accordingly, the Shepard plot of this model shows small scatter around the fitted line; thus, original dissimilarities are well preserved in the reduced number of dimensions (Appendix S1). The NMDS model showed a hierarchical clustering that clearly discriminates between two periods that approximately correspond to austral ‘summer’ (October, November, December, January, February, March, and April) and austral ‘winter’ (May, June, July, August, and September) seasons (Figure 2a). This temporal organization in two periods was completely based on the food web properties and not on the climatic seasonality.

The two resulting periods (referred here as ‘summer’ and ‘winter’) differed substantially between the 16 food web properties employed in the NMDS analysis (Figure 2b), and this was particularly true for the main structural descriptors of network complexity (values of the food web descriptors are available as supporting information; Table S2). The largest differences between ‘summer’ and ‘winter’ were observed for the total number of links and the average links per node (Figure 2b), where values were on average 44% and 26% higher in ‘summer’ than in ‘winter’ (Kruskal–Wallis *p* < .005 in both cases). The number of nodes and connectance exhibited a smaller increase of 15.3% and 9.1% during ‘summer’, respectively (*p* < .042 in both cases), indicating that the depletion of trophic interactions during ‘winter’ was also accompanied by a reduction in the number of species and, consequently, network size.

**Figure 3:**
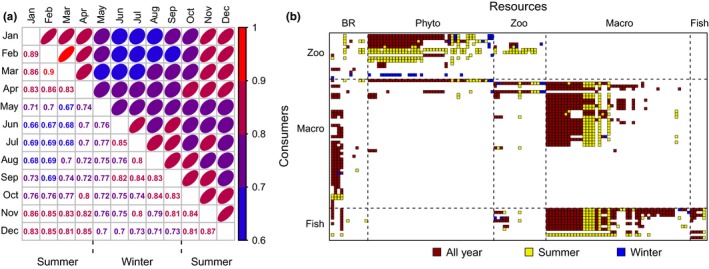
(a) Similarity matrix between food webs obtained for each month. For each pairwise comparison, similarity was estimated as 1—Sorensen index and its strength ranged between 0.64 (weak in blue) and 0.9 (strong in red). Note that the distribution of this index closely corresponds to the summer/winter aggregate distribution obtained in the NMDS ordination (Figure 2). (b) The Peri Food web discriminating between interactions observed only during summer months, only during winter months or across the two seasons, for the following categories: basal resources (BR), phytoplankton (Phyto), zooplankton (Zoo), macroinvertebrates (Macro), and fish (Fish). Rows and columns with zero interactions were removed for clarity

**Figure 4:**
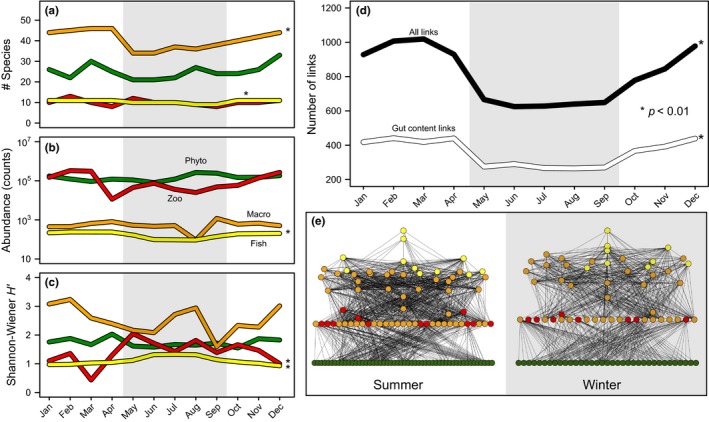
Annual variation in (a) species richness; (b) density [phytoplankton and zooplankton (ind/ml), macroinvertebrates (ind/0.5 m^2^), fish (ind/100 m^2^)]; and (c) diversity of phytoplankton, zooplankton, macroinvertebrates, and fish; and (d) in the number of trophic interactions of the Peri Lake (black line = all the links, including these obtained from the literature; white line = links obtained by gut content analysis). Food web properties can aggregate into two groups of months corresponding to summer and winter (see Results) that are shown, respectively, in white and gray background. Asterisks (*) denote significant differences between summer and winter based on Kruskal–Wallis rank‐sum test. (e) Representative summer (February) and winter (August) food webs, with nodes colored as in (a–c). Note that during winter the food web has fewer links and macroinvertebrates acting as intermediate species

With regard to the identity of trophic interactions, the similarity between networks estimated as (1—Sørensen dissimilarity index) also showed a clear dichotomy throughout the year (Figure 3a). Within ‘summer’ similarity values ranged between 0.9–0.81, whereas in ‘winter’ values were slightly lower in the order of 0.71–0.85. Conversely, the similarity of 0.66–0.69 between months constituting the peak of ‘summer’ (January to March) versus ‘winter’ (May to August) encompassed the lowest values observed in our sample. Using an incomplete dataset to estimate properties for the whole period might produce homogenizing of similarity values. Nevertheless, this analysis complemented the NMDS and showed that the temporal organization detected with the ordination model actually reflected consistency in the identity of trophic interactions within ‘summer’ and ‘winter’. Thus, proposed trophic interactions can be partitioned into three major groups: those that were observed throughout the year, those observed only during ‘summer’, and those present only during ‘winter’ (Figure 3b). This partition not only allows us to identify the core of trophic interactions that maintain the overall integrity of the food web along the year, but also shows that the food web observed in the ‘winter’ constitutes an impoverished version of the food web during ‘summer’ (Figure 4d,e). Besides, the reduction in the total number of links reflected the same pattern than in those obtained by the gut content analysis (Figure 4d). Furthermore, links obtained by the gut content analysis represented between 39% and 46% of the total food web throughout the year (Fig. S2). Therefore, while it is true our methodology neglected intraspecific dietary shifts in some groups, it was relatively representative of the actual pattern.

### 3.2 Seasonal patterns in diversity

The number of trophospecies (nodes) remained relatively constant across months and differed substantially between groups (Figure 4a), with macroinvertebrates exhibiting the highest diversity of species (40.5 ± 4.6 spp, range 34–46), followed by phytoplankton (25.1 ± 3.6 spp, range 21–33), zooplankton (10.1 ± 1.4 spp, range 8–13), and fish (10.4 ± 0.8 spp, range 9–11). The Kruskal–Wallis rank‐sum test suggests that there were no differences in the number of phytoplankton and zooplankton species (*p* > .09 in both cases) between ‘summer’ and ‘winter’ (from October–April and May–September, respectively; see analyses below), but the number of macroinvertebrates and fish species was significantly higher during ‘summer’ (*p* < .004 in both cases). In addition, results from interannual variation in phytoplankton and fish species composition did not show dramatic changes between years (Table S3 and S4). Similarity values ranged 0.6–0.9 and 0.8–1 for phytoplankton and fish composition throughout the sampling period, respectively.

Fish density estimated as the total across all species exhibited a significant seasonal pattern, decreasing from a monthly average count of 219 ± 22 individuals/ 100 m^2^ in ‘summer’ to 120 ± 34 individuals/ 100 m^2^ during ‘winter’ (*p* = .0045; Figure 4). In contrast, no seasonal differences in density were detected for the remaining groups (phytoplankton, zooplankton, macroinvertebrates), even though this trend was nearly significant for zooplankton (*p* = .062).

Seasonal variation in diversity quantified as H^′^, which includes the impact of changes in relative density across months, did not always track observed changes in the number of trophospecies (Figure 4). Employing this index, differences in diversity between ‘summer’ and ‘winter’ months were apparent for both zooplankton (Kruskal–Wallis *p* = .0028) and fish (*p* = .004). Conversely, no clear seasonal trends were detected for phytoplankton (*p* = .09) and macroinvertebrates (*p* = .12).

### 3.3 Environmental correlates

Of all the environmental factors and diversity values fitted (Figure 2a), the littoral alkalinity, water temperature, oxygen concentration, chlorophyll a concentration, macroinvertebrate richness, and fish density showed a gradient that was highly correlated with the ordination (*p* < .05 in all cases; see Appendix S1). Overall, these results are in agreement with the general seasonal patterns in diversity described above, which suggest that the number of species of macroinvertebrate and fish density exhibits a strong seasonal pattern.

## 4 Discussion

Here, we report patterns of variation in community structure and food webs topology throughout the annual cycle. Despite the limitations of our approach (our food web combines data from different and often nonoverlapping sampling periods, see Methods), our analyses showed a strong dichotomous pattern throughout the year in the dataset. The pronounced differences between ‘summer’ and ‘winter’ detected with two alternative analytical approaches (studying topology and complexity of the food webs, Figures 2 and 3), and the strong correlation between the structural patterns detected across the two periods and environmental correlates quantified independently during a time span of 6 years (Figure 2), support the robustness of our results. Although NMDS analysis has been widely used to assess food web structure in freshwater ecosystems (e.g., Thomas et al., 2016, Schmid‐Araya et al., 2016), combining this approach with food web topology tracking is novel. Our results demonstrate that combining both approaches can give new insights into the seasonal differences in qualitative webs. However, more work needs to be done across a variety of systems to ensure the validity of this approach.

Reduction in the number of nodes and links was closely related with the topological simplification of the food web when several descriptors were included in the analysis. Nevertheless, not all the descriptors were equally explanatory. Our analyses showed that temporal variability of the food web is not immediately apparent in analyses of diversity per se but can be identified by the addition of further measures of structure (Figure 4). In the same vein, our results suggest that some descriptors of network structure, such as the total number of trophic interactions, are more sensitive and reliable to study seasonal variation in network structure (Figure 2) than widely employed descriptors such as connectance and network size (see also Martinez, 1992; Riede et al., 2010). Nevertheless, some of the descriptors that we used may be system‐dependent and generalization should be undertaken with caution (Riede et al., 2010). Analyses also indicate that the food web during ‘winter’ represents a simplified version of observed trophic interactions during ‘summer’ (Figure 3).

The NMDS in combination with effect size comparisons across the 16 network descriptors (Figure 2) encapsulates how temporal changes in the community affect network structure. The number of trophic interactions increases by 44.3% between ‘winter’ and ‘summer’, with monthly averages of, respectively, 641.6 and 926.4 interactions, which contrasts with the rise in species number that never surpasses 17.3% when all groups are pooled. Due to the concomitant increase in the number of trophic interactions and network size, connectance was only 9.1% higher during ‘summer’ (Figure 2). Despite a relatively constant connectance of 9.3 ± 0.7% (±*SD*), the variation in the number of trophic interactions per species (i.e., links per node) suggests that the contribution of highly connected species increases during ‘summer’. These species are primarily macroinvertebrates (Figures 2 and 4) and, as described in other systems (Brönmark & Hansson, 2005; Lancaster & Downes, 2013), encompass several univoltine or semivoltine insect species with terrestrial adult stages (i.e., Chironomidae, Trichoptera, Ephemeroptera, Odonata). Therefore, underlying biology (species with semi‐voltine life cycles) may partly explain the contrasting temporal differences in the number of macroinvertebrate species (Figure 4). In contrast, differences observed in fish species richness are unlikely to be meaningful because they have multivoltine life cycles and so probably persist in the lake. In addition, the two fish species that were not captured during some ‘winter’ months, *Awaous tajasica* and *Hoplias lacerdae*, exhibited the lowest density of all species along the year (0.33 and 0.27 ind/100 m^2^, on average, in contrast with a relative density for other species ranging from 1.15 to 12.9 ind/100 m^2^). Macroinvertebrates are present throughout the food web as top predators, intermediate, and basal consumers (Merritt and Cummins 1996, Lancaster & Downes, 2013). Therefore, as we expected, despite the reduction in richness of macroinvertebrates, we did not find significant change in the percentage of intermediate species between seasons (Figure 2b). Regardless of the lake being a relatively closed system, these macroinvertebrate nodes are naturally removed from the food web during the egg or terrestrial stage, as evidenced by the decrease in species number during winter (Figure 4).

Our results also suggest a set of concerted seasonal responses. High temperatures during summer are correlated with a higher primary productivity (chlorophyll a), as well as increased diversity of macroinvertebrates and higher fish density (Figure 2). This suggests that, during winter, the drop in fish density to 54.5% of summer levels may be partly attributed to lower productivity concomitantly with the simplification of the food web as macroinvertebrate species become scarcer (bottom‐up control; see Worm & Myers, 2003; Bailey, Ruhl, & Smith, 2006; Pitois, Lynam, Jansen, Halliday, & Edwards, 2012). This remains speculative, however, because causality cannot be established from our correlational approach. Nevertheless, as it has been described in temperate lakes (Huss, Byström, & Persson, 2008; Persson, Byström, & Wahlström, 2000), the reduction in macroinvertebrates as available resource promotes body size‐dependent intercohort competitive interactions in fish populations, resulting in reductions of fish recruits. Whereas the marginally significant increase in zooplankton density during summer (see 3) seems to support our interpretations, there were no clear seasonal differences in both phytoplankton and macroinvertebrates (Figure 2). Nonetheless, in Lake Constance, even though biomass rather than density was used, and so a quantitative model was produced, top‐down control gives rise to very similar seasonal trends to those reported here in which phytoplankton biomass remains constant or even decreases during late spring and early summer while zooplankton increases (Boit et al., 2012; Gaedke, Hochstädter, & Straile, 2002; Tirok & Gaedke, 2007).

In summary, our results provide a thorough description of how environmental variables, community and food web structure and ecosystem function change in tandem throughout the year in this study system. Due to its simpler composition during winter, the Peri Lake community seems to be overall more vulnerable in this particular season. Given the relatively predictable seasonal changes in photoperiod, temperature and, consequently, primary productivity, we speculate that this conclusion may be applicable to other lake systems with a similar community structure. Increasing environmental change is occurring globally in lakes (O'Reilly et al., 2015; Schneider & Hook, 2010), and so identifying and monitoring the season in which the food web is simplest may be particularly relevant for conservation purposes and to understand how lake systems might evolve.

## Conflict of interest

None declared.
