# Supplementary material for: Winter is coming: Food web structure and seasonality in a subtropical freshwater coastal lake
Source: Ecol Evol. 2017 May 17;7(13):4534–42. doi: 10.1002/ece3.3031 (PMC5496567; doi:10.1002/ece3.3031)
Supplement: Supplementary file 1 [file ECE3-7-4534-s001.docx]

**Supporting information for**

**Winter is coming: food web structure and seasonality in a subtropical freshwater coastal-lake.**

I. Peralta-Maraver, A. Robertson, E. L. Rezende, A. L. Lemes-Silva, D. Tonetta, M. Lopes, R. Schmitt, N. K. Leite, A. Nuñer, M. M. Petrucio.

**Index**

Appendix S1 (page 2)

- Identified organisms

- Shepard plot of the NDMS ordination.

- Fitting coefficients of environmental factors in the NDMS ordination model.

- Diet references list.

Table S1 Environmental factors (page 7)

Table S2 Food web properties (page 9)

Table S3 Similarity matrices at different years for composition of phytoplankton community (1 – Sorensen index) (page 11)

Table S4 Similarity matrices at different years for composition of fish community (1 – Sorensen index) (p11)

Fig S1 Sampling schedule (page 12)

Fig S2 Number and proportion of links extracted from the literature (white) and the gut content analysis (grey) throughout the year. (page 12)

**Appendix S1**

**Shepard plot of the NDMS ordination.** Scatter around the regression of distances between each pair of communities against their original dissimilarities. The fit is shown as a monotone step line.

**
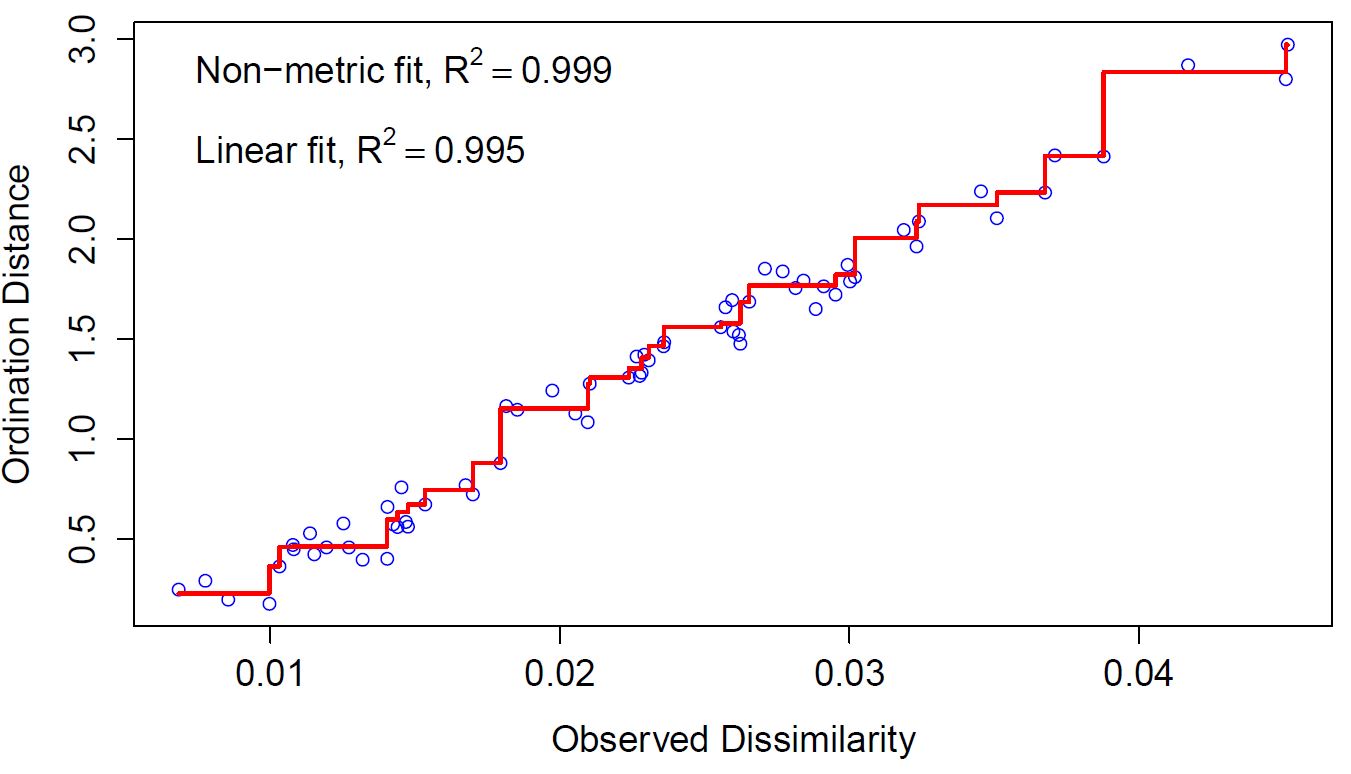
**

**Fitting coefficients of environmental factors, abundance and species richness against the NDMS ordination model.**

|  | **NMDS1** | **NMDS2** | **r2** | **P** |
| --- | --- | --- | --- | --- |
| Lit.Conductivity | -0.6659 | 0.7460 | 0.3473 | 0.147 |
| Lit.pH | -0.6526 | -0.7577 | 0.0694 | 0.730 |
| Lit.Dis.Org.Carb | 0.9992 | -0.0390 | 0.2717 | 0.240 |
| Lit.Water.Temp | -0.8898 | -0.4563 | 0.8294 | 0.001* |
| Lit.Total.P | -0.6533 | 0.7571 | 0.2315 | 0.327 |
| Lit.Dis.Oxy | 0.9058 | 0.4236 | 0.6188 | 0.007* |
| Lit.Wind.Speed | -0.6202 | 0.7844 | 0.3790 | 0.144 |
| Lit.Transparency | -0.2542 | 0.9672 | 0.0867 | 0.665 |
| Lit.Total.N | 0.9140 | 0.4057 | 0.2854 | 0.254 |
| Lit.Chlorophyll | -0.4657 | -0.8849 | 0.6039 | 0.011* |
| Lit.Alkalinity | -0.9566 | 0.2913 | 0.5431 | 0.038* |
| N.P | 0.1620 | 0.1620 | 0.3170 | 0.165 |
| Rainfall | -0.9927 | -0.1209 | 0.2272 | 0.325 |
| Water.level | -0.8796 | -0.4758 | 0.2213 | 0.368 |
| log10(dens.phyto) | -0.1354 | 0.9908 | 0.0862 | 0.661 |
| log10(dens.zoo) | -0.9597 | -0.2812 | 0.3123 | 0.190 |
| log10(dens.macro) | -0.4404 | -0.8978 | 0.0584 | 0.730 |
| log10(dens.fish) | -0.9495 | -0.3138 | 0.7118 | 0.004* |
| richness.phyto | -0.9524 | 0.3050 | 0.3972 | 0.109 |
| richness.zoo | -0.5458 | 0.8379 | 0.1209 | 0.544 |
| richness.macro | -0.7900 | -0.6131 | 0.9528 | 0.001* |

**List of identified organisms (nodes).**

PHYTOPLANKTON

*Actinastrum aciculare*

*Anabaena* sp.

*Ankistrodesmus* sp.

*Aphanocapsa conferta*

*Aphanothece* sp.

*Aulacoseira ambigua*

*Botryococcus braunii*

*Chlorella* sp.

Chroococales

*Closterium* sp.

*Cosmarium portianum*

*Cosmarium* sp. 1

*Cosmarium* sp. 2

*Cylindrospermopsis raciborskii*

*Dictyosphaerium ehrenbergianum*

*Encyonema* sp.

*Fragilaria capucina*

*Fragillaria* sp.

*Golenkinia pauscispina*

*Golenkinia radiata*

*Limnothrix redekei*

*Monoraphidium arcuatum*

*Monoraphidium irregulare*

*Nephrochlamys* sp.

*Oocystis lacustris*

*Pediastrum* sp.

*Peridiniopsis* sp.

*Peridinium* sp.

*Pinnularia* sp.

*Planktolyngbya circumcreta*

*Planktolyngbya limnetica*

*Planktolyngbya* sp.

*Pseudanabaena catenata*

*Scenedesmus oahuensis*

*Scenedesmus* sp. 1

*Scenedesmus* sp. 2

*Scenedesmus spinosus*

*Staurastrum* sp. 1

*Staurastrum* sp. 2

*Staurastrum tetracerum*

*Staurodesmus cuspidatus*

*Tetraëdron caudatum*

*Trachelomonas* sp.

*Urosolenia* sp.

ZOOPLANKTON

*Asplanchna* sp.

Bdelloidea

*Bosmina freyi*

*Bosmina hagmanni*

*Brachionus angularis*

*Brachionus calyciflorus*

*Collotheca* sp.

Copepoda larvae

*Filinia longiseta*

Harpacticoida

*Hexarthra intermedia*

Nauplio

*Polyarthra libera*

*Polyarthra vulgaris*

*Thermocyclops minutus*

*Trichocerca capucina*

*Trichocerca dixon*

*Trichocerca similis*

MACROINVERTEBRATES

*Ablabesmyia* sp.

Aeshnidae

*Aphylla* sp.

Bivalve

*Caladomyia ortoni*

*Chaoborus* sp.

*Chironomus riparius*

*Cladopelma forcipis*

*Coelotanypus* sp.

Coenagrionidae

*Cryptochironomus* sp.

*Cyrnellus* sp.

*Cytheridella ilosvayi*

*Djalmabatista* sp.

Dytiscidae

Elmidae

*Endotribelos* sp.

*Fittkauimyia* sp.

Gastropoda

*Goeldchironomus maculatus*

Gyrinidae

*Hetaerina* sp.

Hidracarina

Hirudinea

*Labrundinia* sp.

Libellulidae

*Limnodrilus hoffmeisteri*

*Limnodrilus* sp.

*Lopescladius* sp.

*Macronema* sp.

Nematoda

*Nilothauma* sp.

Odontoceridae

*Oecetis* sp.

Oligochaeta

*Pelomus psamorphilos*

*Phyllocycla* sp.

*Polipedilum* sp. 1

*Polypedilum* sp. 2

*Progomphus* sp.

*Psoletra rufa*

Smicridea

*Stempellina* sp.

*Stenochironomus* sp.

*Stenocypris major*

Tanaidacea

*Tauriphila* sp.

FISH

*Awaous tajasica*

*Centropomus parallelus*

*Geophagus brasiliensis*

*Hoplias lacerdae*

*Hoplias malabaricus*

*Hyphessobrycon luetkeni*

*Lycengraulis grossidens*

*Odontesthes argentinensis*

*Platanichthys platana*

*Rhamdia quelen*

*Tilapia rendali*

**Diet reference list.**

Bleiwas, A. H. and P. M. Stokes. 1985. Collection of large and small food particles by Bosmina. Limnol. Oceanogr. 30:1090–1092.

Bogdan, K. G. and J. J. Gilbert. 1982. Seasonal patterns of feeding by natural populations of Keratella, Polyarthra, and Bosmina: Clearance rates, selectivities, and contributions to community grazing. Limnol. Oceanogr. 27:918–934.

Bradley, M. D. K. and J. D. Reynolds. 1987. Diet of the leeches Erpobdella octoculata (L) and Helobdella stagnalis (L) in a lotic habitat subject to organic pollution. Freshwater Biol. 18:267–275.

Burcher, C. L. and L. A. Smock. 2002. Habitat distribution, dietary composition and life history characteristics of odonate nymphs in a blackwater coastal plain stream. Am. Midl. Nat. 148:75–89.

Castilho–Noll, M. S. M. and M. S. Arcifa. 2007. Chaoborus diet in a tropical lake and predation of microcrustaceans in laboratory experiments. Acta Limnol. Bras. 19:163–174.

De Manuel Barrabin, J. 2000. The rotifers of Spanish reservoirs: Ecological, Systematical and zoogeographical remarks. Limnetica 19:91–167.

Dumont, H. J. et al. 2012. Intrazooplankton predation (Vol. 60). Springer Science and Business Media.

Elliott, J. M. 2006. Critical periods in the life cycle and the effects of a severe spate vary markedly between four species of elmid beetles in a small stream. Freshwater Biol. 51:1527–1542.

Elliott, J. M. 2008. The ecology of Riffle Beetles (Coleoptera: Elmidae). Freshwater. Rev. 1:189–203.

Flint, O. S. Jr. 1991. Studies of neotropical caddisflies, XLV: The taxonomy, phenology and faunistics of the Trichoptera of Antioquia, Colombia. Smithson. Contr. Zool.
 52:1–113.

Friday, L. E. 1988. A key to the adults of British water beetles. Field Stud 7:151.

Gilljam, D. et al. 2011. Seeing Double: Size–Based and Taxonomic Views of Food Web Structure. Adv. Ecol. Res. 45:67–133.

Johansson, A. and A. N. Nilsson. 1992. Dytiscus Zatissimus and D. circumcinctus (Coleoptera, Dytiscidae) larvae as predators on three case–making caddis larvae. Hydrobiologia 248:201–213.

Lancaster, J. et al. 2005. Intraguild omnivory in predatory stream insects. J. Anim. Ecol.74:619–629.

Layer, K. et al. 2010. Food Web Structure and Stability in 20 Streams Across a Wide pH Gradient. Adv. Ecol. Res. 42:265–299.

Ledger, M. E. et al. 2013. Drought alters the structure and functioning of complex food webs. Nat. Clim. Chang. 3:223–227.

Oganjan, K. et al. 2013. Food spectrum of the omnivorous rotifer Asplanchna priodonta in two large northeastern European lakes of different trophy. Oceanol. Hydrobiol. Stud. 42:314–323.

Reiso, R. and L. E. Brittain. 2000. Life cycle, diet and habitat of Polvcentropus flavomaculatus, Plectrocnemia conspersa and Rhyacophila nubila (Trichoptera) in Øvre Heimdalen, Jotunheimen Mountains, Norway. Nor. J. Entomol. 47:113–124.

Rundle S.D. et al. 2002. Freshwater meiofauna. Biology and Ecology. Backhuys Publishers.

Sarma, S. S. S. et al. 1998. Feeding preference and population growth of Asplanchna brightwelli (Rotifera) offered two non–evasive prey rotifers. Hydrobiologia 361:77–87.

Shuhaimi–Othman, M. et al. 2011. Toxicity of metals to a freshwater ostracod: Stenocypris major. J Toxicol. 2011:1–8.

Slack, H. D. 1936. The food of caddis fly (Trichoptera) larvae. J. Anim. Ecol.74:105–115.

Tachet, H. et al. 2002. Invertebratébrés d’eau douce. Systematique, biologie, écologie. CNRS Editions.

Tate, A. W. and A. E. Hershey. 2003. Selective feeding by larval dytiscids (Coleoptera: Dytiscidae) and effects of fish predation on upper littoral zone macroinvertebrate communities of arctic lakes. Hydrobiologia 497:13–23.

Tavares, A. F. and D. D. Williams. 1990. Life histories, diet, and niche overlap of three sympatric species of Elmidae Coleoptera in a temperate stream. Can. Entomol. 122:563–577.

Thorp, J. H. and A. P. Covich. 2009. Ecology and classification of North American freshwater invertebrates. Academic press.

Townsend, C. R. and A. G. Hildrew. 1979 Resource Partitioning by Two Freshwater Invertebrate Predators with Contrasting Foraging Strategies. J. Anim. Ecol. 48:909–920.

Vaughn, C. C. and C. C. Hakenkamp. 2001. The functional role of burrowing bivalves in freshwater ecosystems. Freshwater Biol. 46:1431–1446.

Warren, P. H. 1989. Spatial and temporal variation in the structure of a freshwater food web. Oikos 55:299–311.

Young, J. O. 1980. A serological investigation of the diet of Helobdella stagnalis (Hirudinea: Glossiphoniidae) in British lakes. Can. J. Zool. 192:467–488.

Young, J. O. et al. 1993. Competitive interactions between the lake–dwelling leeches Glossiphonia complanata and Helobdella stagnalis: an experimental investigation of the significance of a food refuge. Oecologia 93:156–161.

**Table S1.** Monthly mean and standard deviation (±SD) of the environmental factors recorded in the Peri Lake during the sampling period (2008-2014). Total nitrogen (Nitrogen, µg L^-1^), total phosphorus (P, µg L^-1^), nitrogen-phosphorus ratio (N:P), dissolved organic carbon (DOC; mg L-1), chlorophyll-a concentration (Chl a, µg L^-1^), dissolved oxygen (DO, mg L^-1^), water temperature (W Temp, ºC), alkalinity (mEq L^-1^), Conductivity (µS cm-1), pH, water transparency (Trans, m), wind velocity (Wind, m s^-1^), rainfall (mm) and water level (W Level, cm).

| **Month** | **n** | **Total N** | **Total P** | **N:P ratio** | **DOC** | **Chl A** | **DO** | **W Temp** | **Alkalinity** | **Conductivity** | **pH** | **Trans** | **Wind** | **Rainfall** | **W level** |
| --- | --- | --- | --- | --- | --- | --- | --- | --- | --- | --- | --- | --- | --- | --- | --- |
|  |  |  |  |  |  |  |  |  |  |  |  |  |  |  |  |
| **Littoral zone** |  |  |  |  |  |  |  |  |  |  |  |  |  |  |  |
| **January** | 252 | 584.97±319.27 | 16.81±1.65 | 49.73±34.53 | 4.71±2.12 | 25.69±13.29 | 7.53±1.03 | 27.42±1.32 | 0.1±0.04 | 75.73±6.92 | 7.15±0.44 | 0.9±0.25 | 2.72±0.9 | 223.2±167.08 | 267.55±24.86 |
| **February** | 252 | 443.83±389.81 | 13.17±5.58 | 47.03±27.17 | 3.84±1.22 | 26.53±13.17 | 7.21±0.7 | 28.5±0.71 | 0.12±0.07 | 72.17±8.28 | 7.34±0.68 | 0.9±0.15 | 2.07±0.9 | 138.99±82.98 | 268.5±23 |
| **March** | 252 | 554.24±328.62 | 13.2±3.11 | 46.83±28.01 | 4.38±0.92 | 27.72±6.74 | 7.61±1.39 | 26.08±1.77 | 0.1±0.05 | 71.67±7.58 | 6.86±0.5 | 0.93±0.2 | 4±0.93 | 175.49±126.4 | 269.17±24.04 |
| **April** | 252 | 733.05±286.24 | 10.77±2.19 | 52.14±27.27 | 4.83±1.16 | 26.18±9.99 | 7.82±0.68 | 24.57±1.3 | 0.09±0.03 | 65.4±12.44 | 6.46±0.65 | 0.89±0.18 | 1.85±0.89 | 113.34±73.96 | 266.01±19.32 |
| **May** | 252 | 698.84±301.06 | 12.21±2.67 | 56.48±32.37 | 5.29±1.22 | 16.52±12.26 | 8.25±0.6 | 20.99±1.6 | 0.09±0.03 | 69.76±5.67 | 6.85±0.36 | 0.97±0.11 | 2.53±0.97 | 109.43±117.29 | 267.63±25.72 |
| **June** | 252 | 620.75±290.14 | 11.22±2.03 | 52.91±34.98 | 5.63±1.22 | 23.79±8.27 | 8.46±1.03 | 18.62±0.84 | 0.08±0.03 | 66.29±5.48 | 7.03±0.72 | 0.94±0.18 | 1.43±0.94 | 71.4±29.56 | 266.41±16.8 |
| **July** | 252 | 739.84±244.98 | 13.39±2.74 | 50.25±26.99 | 5.56±1.57 | 17.72±4.11 | 8.58±0.87 | 18±1.76 | 0.09±0.03 | 67.48±5.97 | 6.63±0.68 | 0.93±0.19 | 2.04±0.93 | 73.8±51.1 | 264.09±13.32 |
| **August** | 252 | 637.88±262.17 | 13.53±3.25 | 66.93±52.93 | 4.04±1.87 | 20.03±5.71 | 8.85±0.83 | 18.61±1.51 | 0.1±0.05 | 71.37±13.52 | 6.8±0.39 | 0.9±0.16 | 1.82±0.9 | 95.8±107.01 | 264.18±13.64 |
| **September** | 252 | 561.34±309.43 | 15±2.38 | 54.8±41.18 | 3.99±1.55 | 18.28±3.45 | 8.31±0.79 | 20.42±1.11 | 0.1±0.05 | 66.83±7.61 | 7.04±0.51 | 0.93±0.14 | 2.87±0.93 | 126.84±77.26 | 268.19±25.42 |
| **October** | 252 | 600.17±322.7 | 10.83±0.77 | 66.15±38.05 | 4.19±1.41 | 18.57±5.93 | 8.51±1.4 | 22.13±1.06 | 0.09±0.05 | 63.56±18.28 | 6.98±0.71 | 0.99±0.17 | 2.08±0.99 | 63.86±33.96 | 270.48±19.5 |
| **November** | 252 | 461.63±199.74 | 12.79±2 | 51.47±42.1 | 4.33±2.35 | 19.34±5.1 | 8.56±0.32 | 24.25±0.96 | 0.1±0.06 | 72.49±6.95 | 7.18±0.61 | 1.06±0.17 | 4.44±1.06 | 79.83±41.21 | 268.13±22.54 |
| **December** | 252 | 469.67±301.74 | 19.33±3.01 | 34.76±26.73 | 4.48±2.23 | 25.14±8.7 | 7.27±0.68 | 25.62±0.78 | 0.1±0.05 | 72.15±6.5 | 7.1±0.69 | 1.05±0.14 | 3.06±1.05 | 92.23±60.55 | 268.75±24.99 |
|  |  |  |  |  |  |  |  |  |  |  |  |  |  |  |  |
| **Pelagic zone** |  |  |  |  |  |  |  |  |  |  |  |  |  |  |  |
| **January** | 336 | 669.67±347.51 | 14.26±3.07 | 49.73±34.53 | 4.46±1.48 | 27.5±15.4 | 7.77±0.97 | 26.9±0.82 | 0.12±0.03 | 75.11±7.39 | 6.86±0.18 | 0.89±0.32 | 5.28±4.05 | 223.2±167.08 | 267.55±24.86 |
| **February** | 336 | 571.54±387.88 | 14.23±5.01 | 47.03±27.17 | 4.25±0.83 | 30.41±15.83 | 6.99±0.82 | 28.07±0.85 | 0.12±0.06 | 71.98±8.34 | 7.15±0.69 | 0.89±0.18 | 2.3±2.15 | 138.99±82.98 | 268.5±23 |
| **March** | 336 | 487.34±266.48 | 12.99±3.12 | 46.83±28.01 | 4.24±0.92 | 29.65±9.86 | 6.91±1.69 | 25.99±1.58 | 0.11±0.05 | 71.21±8.9 | 6.89±0.53 | 0.93±0.16 | 6.22±2.69 | 175.49±126.4 | 269.17±24.04 |
| **April** | 336 | 659.09±347.18 | 14.3±5.18 | 52.14±27.27 | 4.3±1.16 | 25.56±7.28 | 7.47±0.74 | 24.29±1.12 | 0.11±0.03 | 67.29±11.07 | 6.43±0.71 | 0.91±0.2 | 3.48±3.14 | 113.34±73.96 | 266.01±19.32 |
| **May** | 336 | 667.38±348.02 | 12.75±3.44 | 56.48±32.37 | 4.3±1.22 | 22.71±14.63 | 8.24±0.34 | 20.57±0.83 | 0.12±0.04 | 67.96±5.61 | 6.74±0.32 | 0.94±0.12 | 4.38±3.29 | 109.43±117.29 | 267.63±25.72 |
| **June** | 336 | 542.63±237.65 | 11.66±2.58 | 52.91±34.98 | 4.16±1.22 | 26.89±13.46 | 8.4±1.09 | 18.43±0.87 | 0.12±0.04 | 65.67±5.72 | 7.01±0.95 | 0.96±0.21 | 2.23±3.2 | 71.4±29.56 | 266.41±16.8 |
| **July** | 336 | 568.28±227.79 | 12.95±3.61 | 50.25±26.99 | 4.42±1.57 | 24.43±11.3 | 8.4±1.09 | 17.82±1.57 | 0.12±0.04 | 67.66±6.3 | 6.71±0.64 | 0.92±0.16 | 1.79±1.96 | 73.8±51.1 | 264.09±13.32 |
| **August** | 336 | 793.04±399.74 | 14.67±6.01 | 66.93±52.93 | 4.21±1.18 | 24.73±11.26 | 8.71±0.79 | 18.22±1.18 | 0.12±0.04 | 72.01±13.37 | 6.83±0.37 | 0.88±0.13 | 3.21±3.49 | 95.8±107.01 | 264.18±13.64 |
| **September** | 336 | 740.53±444.52 | 15.52±6.05 | 54.8±41.18 | 4.33±0.93 | 23.05±9.11 | 8.28±0.55 | 20.22±1.15 | 0.11±0.05 | 66.14±7.24 | 6.91±0.28 | 1±0.14 | 3.25±2.62 | 126.84±77.26 | 268.19±25.42 |
| **October** | 336 | 612.98±223.65 | 10.07±2.41 | 66.15±38.05 | 4.02±1.47 | 24.63±13.24 | 7.99±0.99 | 21.93±1.25 | 0.11±0.05 | 64.04±17.94 | 7.15±0.58 | 1±0.13 | 2.82±2 | 63.86±33.96 | 270.48±19.5 |
| **November** | 336 | 510.69±244.35 | 12.49±4.14 | 51.47±42.1 | 5.47±3.11 | 23.18±9.09 | 8.26±0.52 | 24.13±0.91 | 0.13±0.04 | 72.42±7.09 | 7.1±0.76 | 1.01±0.14 | 2.67±3.74 | 79.83±41.21 | 268.13±22.54 |
| **December** | 336 | 458.99±267.82 | 16.48±4.79 | 34.76±26.73 | 4.56±1.55 | 27.34±10.5 | 7.32±0.73 | 25.47±1.24 | 0.11±0.05 | 72.78±6.44 | 7.1±0.58 | 1.02±0.12 | 5.26±3.28 | 92.23±60.55 | 268.75±24.99 |

**Table S2. Food web properties and diversity values.** Monthly values of food web properties [S (number of trophospecies), L (number of links), L/S (link density), C (connectance, L/S2), FracTop (fraction of nodes acting as consumer), FracIntermed (fraction of nodes acting as consumer and resource), FracBasal (fraction of nodes that only act as resource), GenSD (standard deviation of the number of resources per node), VulSD (standard deviation of the number of consumer per node), MeanSWTL (mean short weighted trophic level), MeanShortChn (mean shorted chain to a basal species), FracOmniv (fraction of consumer nodes that consume of resources from more than on trophic level), FracHerb (fraction of nodes than only consume basal resources), FracCannibal (fraction of cannibal consumer), ResourceCount (total number of resources nodes), ConsumerCount (total number of consumer nodes)] and mean ± SD (credible intervals) of biological diversity calculated with the Shannon-Wiener’s index (DivPhyto: phytoplankton diversity; DivZoo: zooplankton diversity; DivMacro: macroinvertebrates diversity, Divfish: fish diversity).

|  | **January** | **February** | **March** | **April** | **May** | **Jun** | **July** | **August** | **September** | **October** | **November** | **December** |
| --- | --- | --- | --- | --- | --- | --- | --- | --- | --- | --- | --- | --- |
| **S** | 97.00 | 104.00 | 105.00 | 97.00 | 95.00 | 81.00 | 85.00 | 86.00 | 84.00 | 91.00 | 99.00 | 103.00 |
| **L** | 928.00 | 1007.00 | 1019.00 | 930.00 | 666.00 | 625.00 | 628.00 | 640.00 | 649.00 | 778.00 | 845.00 | 978.00 |
| **L/S** | 9.60 | 10.09 | 9.70 | 9.80 | 7.36 | 7.90 | 7.54 | 7.44 | 7.83 | 9.04 | 9.01 | 10.06 |
| **C** | 0.10 | 0.10 | 0.09 | 0.10 | 0.08 | 0.10 | 0.09 | 0.09 | 0.09 | 0.10 | 0.09 | 0.10 |
| **FracTop** | 0.07 | 0.07 | 0.07 | 0.08 | 0.08 | 0.11 | 0.07 | 0.07 | 0.07 | 0.09 | 0.07 | 0.06 |
| **FracIntermed** | 0.57 | 0.57 | 0.54 | 0.57 | 0.57 | 0.56 | 0.55 | 0.52 | 0.55 | 0.55 | 0.55 | 0.54 |
| **FracBasal** | 0.36 | 0.37 | 0.39 | 0.35 | 0.35 | 0.33 | 0.38 | 0.41 | 0.38 | 0.36 | 0.38 | 0.40 |
| **FracOmniv** | 0.46 | 0.43 | 0.46 | 0.41 | 0.43 | 0.49 | 0.42 | 0.40 | 0.42 | 0.45 | 0.47 | 0.43 |
| **FracCanibal** | 0.05 | 0.05 | 0.06 | 0.05 | 0.06 | 0.07 | 0.05 | 0.06 | 0.06 | 0.05 | 0.05 | 0.06 |
| **FracHerbiv** | 0.28 | 0.27 | 0.25 | 0.28 | 0.31 | 0.23 | 0.25 | 0.24 | 0.25 | 0.27 | 0.26 | 0.25 |
| **GenSD** | 1.20 | 1.24 | 1.25 | 1.21 | 1.31 | 1.09 | 1.23 | 1.23 | 1.20 | 1.23 | 1.30 | 1.31 |
| **VulSD** | 1.00 | 0.96 | 1.00 | 1.12 | 1.13 | 1.14 | 1.03 | 1.03 | 1.05 | 0.97 | 1.05 | 1.00 |
| **MeanSWLT** | 1.88 | 1.88 | 1.86 | 1.90 | 1.86 | 1.96 | 1.87 | 1.84 | 1.83 | 1.90 | 1.86 | 1.83 |
| **MeanShortChn** | 1.72 | 1.73 | 1.70 | 1.74 | 1.71 | 1.80 | 1.72 | 1.70 | 1.63 | 1.76 | 1.72 | 1.70 |
| **ResourceCount** | 90.00 | 97.00 | 98.00 | 89.00 | 87.00 | 72.00 | 79.00 | 80.00 | 78.00 | 83.00 | 92.00 | 97.00 |
| **ConsumerCount** | 62.00 | 66.00 | 64.00 | 63.00 | 62.00 | 54.00 | 53.00 | 51.00 | 52.00 | 58.00 | 61.00 | 62.00 |
| **DivPhyto** | 1.758±0.003 | 1.880±0.003 | 1.667±0.005 | 2.032±0.004 | 1.616±0.004 | 1.580±0.005 | 1.670±0.003 | 1.649±0.002 | 1.717±0.002 | 1.549±0.003 | 1.872±0.003 | 1.828±0.003 |
|  | (1.751-1.764) | (1.874-1.887) | (1.658-1.676) | (2.025-2.038) | (1.609-1.623) | (1.571-1.59) | (1.663-1.677) | (1.645-1.653) | (1.713-1.721) | (1.543-1.555) | (1.866-1.878) | (1.823-1.833) |
| **Div Zoo** | 1.103±0.003 | 1.358±0.002 | 0.436±0.002 | 1.295±0.009 | 2.048±0.004 | 1.712±0.003 | 1.426±0.005 | 1.813±0.005 | 1.39±0.004 | 1.663±0.003 | 1.466±0.003 | 1.006±0.002 |
|  | (1.097-1.109) | (1.354-1.362) | (0.4315-0.440) | (1.277-1.313) | (2.041-2.056) | (1.706-1.717) | (1.415-1.436) | (1.804-1.822) | (1.381-1.398) | (1.656-1.67) | (1.46-1.471) | (1.002-1.011) |
| **DivMacro** | 3.081±0.061 | 3.237±0.051 | 2.594±0.063 | 2.396±0.061 | 2.167±0.074 | 2.085±0.079 | 2.718±0.059 | 2.944±0.111 | 1.598±0.056 | 2.332±0.0701 | 2.277±0.068 | 3.014±0.060 |
|  | (2.959-3.195) | (3.136-3.334) | (2.471-2.718) | (2.277-2.513) | (2.026-2.314) | (1.932-2.239 | (2.601-2.832) | (2.723-3.151) | (1.493-1.712) | (2.192-2.469) | (2.143-2.41) | (2.896-3.126) |
| **DivFish** | 0.975±0.009 | 0.982±0.008 | 1.028±0.008 | 1.049±0.009 | 1.122±0.01 | 1.32±0.012 | 1.33±0.012 | 1.317±0.013 | 1.142±0.011 | 1.065±0.009 | 1.012±0.009 | 0.928±0.009 |
|  | (0.958-0.975) | (0.967-0.982) | (1.012-1.028) | (1.032-1.049) | (1.102-1.121) | (1.295-1.320) | (1.306-1.330) | (1.292-1.317) | (1.121-1.142) | (1.048-1.065) | (0.993-1.012) | (0.910-0.928) |

**Table S3. Phytoplankton community composition throughout the sampling period.**

| **Year** | **2009** | **2010** | **2011** | **2012** | **2013** | **2014** |
| --- | --- | --- | --- | --- | --- | --- |
| **2009** | 1 |  |  |  |  |  |
| **2010** | 0.60 | 1 |  |  |  |  |
| **2011** | 0.70 | 0.70 | 1 |  |  |  |
| **2012** | 0.70 | 0.70 | 0.70 | 1 |  |  |
| **2013** | 0.70 | 0.70 | 0.70 | 0.60 | 1 |  |
| **2014** | 0.70 | 0.70 | 0.60 | 0.60 | 0.80 | 1 |

**Table S4. Fish community composition throughout the sampling period.**

| **Year** | **2008** | **2009** | **2010** | **2011** |
| --- | --- | --- | --- | --- |
| **2008** | 1.00 |  |  |  |
| **2009** | 1.00 | 1.00 |  |  |
| **2010** | 0.90 | 1.00 | 1.00 |  |
| **2011** | 0.90 | 0.80 | 0.80 | 0.80 |


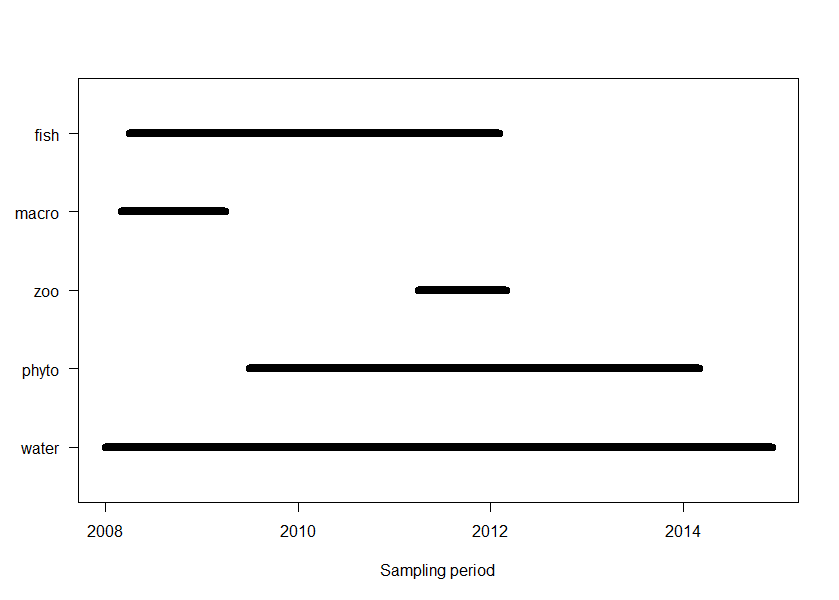
**Fig. S1. Sampling schedule.**

**Fig. S2. Number and proportion of links extracted from the literature (white) and the gut content analysis (grey) throughout the year.** Note that both kinds of links follow the same pattern.
